# N/S Co-doped Carbon Derived From Cotton as High Performance Anode Materials for Lithium Ion Batteries

**DOI:** 10.3389/fchem.2018.00078

**Published:** 2018-04-26

**Authors:** Jiawen Xiong, Qichang Pan, Fenghua Zheng, Xunhui Xiong, Chenghao Yang, Dongli Hu, Chunlai Huang

**Affiliations:** ^1^Guangzhou Key Laboratory for Surface Chemistry of Energy Materials, New Energy Research Institute, School of Environment and Energy, South China University of Technology, Guangzhou, China; ^2^Guangdong Engineering and Technology Research Center for Surface Chemistry of Energy Materials, New Energy Research Institute, School of Environment and Energy, South China University of Technology, Guangzhou, China; ^3^Jiangsu Key Lab of Silicon Based Electronic Materials, Jiangsu GCL Silicon Material Technology Development Co., Ltd, Xuzhou, China

**Keywords:** lithium-ion batteries, anode materials, sustainable, cotton, N/S co-doped carbon

## Abstract

Highly porous carbon with large surface areas is prepared using cotton as carbon sources which derived from discard cotton balls. Subsequently, the sulfur-nitrogen co-doped carbon was obtained by heat treatment the carbon in presence of thiourea and evaluated as Lithium-ion batteries anode. Benefiting from the S, N co-doping, the obtained S, N co-doped carbon exhibits excellent electrochemical performance. As a result, the as-prepared S, N co-doped carbon can deliver a high reversible capacity of 1,101.1 mA h g^−1^ after 150 cycles at 0.2 A g^−1^, and a high capacity of 531.2 mA h g^−1^ can be observed even after 5,000 cycles at 10.0 A g^−1^. Moreover, excellently rate capability also can be observed, a high capacity of 689 mA h g^−1^ can be obtained at 5.0 A g^−1^. This superior lithium storage performance of S, N co-doped carbon make it as a promising low-cost and sustainable anode for high performance lithium ion batteries.

## Introduction

In the past decade, lithium-ion batteries (LIBs) have been widely used as power sources for computing, communications, consumer batteries (3C battery), and electric vehicle. And LIBs with a wide range of applications which due to its have high working voltage, high energy density, and long service life (Goodenough and Kim, 2010; Goodenough and Park, 2013; Zheng et al., 2015). Currently, graphite is mostly used as anode material for commercial LIBs due to its good electronic conductivity, low cost and outstanding cycling stability. However, graphite can not meet the increased energy and power density of high performance LIBs due to the low specific capacity and poor rate performance (Huang et al., 2016; Wang et al., 2017; Pan et al., 2018). On the other hand, the low lithiation/de-lithiation potentials (< 0.3 V vs. Li^+^/Li) which resulting in seriously security issue (Guo et al., 2011). Therefore, it is necessary to develop novel anode materials to replace graphite anode for high-performance LIBs (Pan et al., 2017a).

Recently, amorphous carbon and hard carbon are attracted attentions as promising anode to replace graphite anode for LIBs, which due to these carbonaceous materials exhibit higher specific capacity and offer higher lithiation/de-lithiation potential (Wang et al., 2009; Casas and Li, 2012; Tang et al., 2012). Moreover, these carbonaceous materials with partially graphitic carbons which can accommodate Li^+^ in the disordered interlayers as well as in the micropores, and can exhibit excellent cycling stability and rate capability (Wu et al., 2003). On the other hand, other carbonaceous materials such as carbon nanotube, graphene and fullerenes were also developed as anode for LIBs, and exhibit excellent electrochemical performance (Etacheri et al., 2015; Wang et al., 2015). However, in order to synthesize these carbonaceous materials which rely on hydrocarbon precursors, resulting in expensive cost and commercially nonviable. Therefore, it is necessary to explore a scalable and inexpensive precursor as carbon sources for these carbonaceous materials as anode materials for LIBs.

Nowadays, multitudinous biomass raw materials have been extensively used as precursor for carbonaceous materials and application in LIBs. So many biomass raw materials attracted attention such as peanut (Ding et al., 2015), ramie (Jiang et al., 2016), sisal (Yu et al., 2015), bamboo (Jiang et al., 2014), green tea leaves (Han et al., 2014), peat moss (Ding et al., 2013), rice husk (Wang et al., 2013), banana peel (Lotfabad et al., 2014), and so on. However, cotton attracted more attention and have been considered as the most promising compared with the other biomass materials due to its abundant and low cost. On the other hand, in China, cotton is planted around 550 Million tons per year and giant cotton-products are abandoned which from clothes, medical alcohol cotton, and so on. Moreover, lots of abandoned cotton-products will bring lots of problems, such as environmental pollution, safe question, and so on.

**Figure 1:**
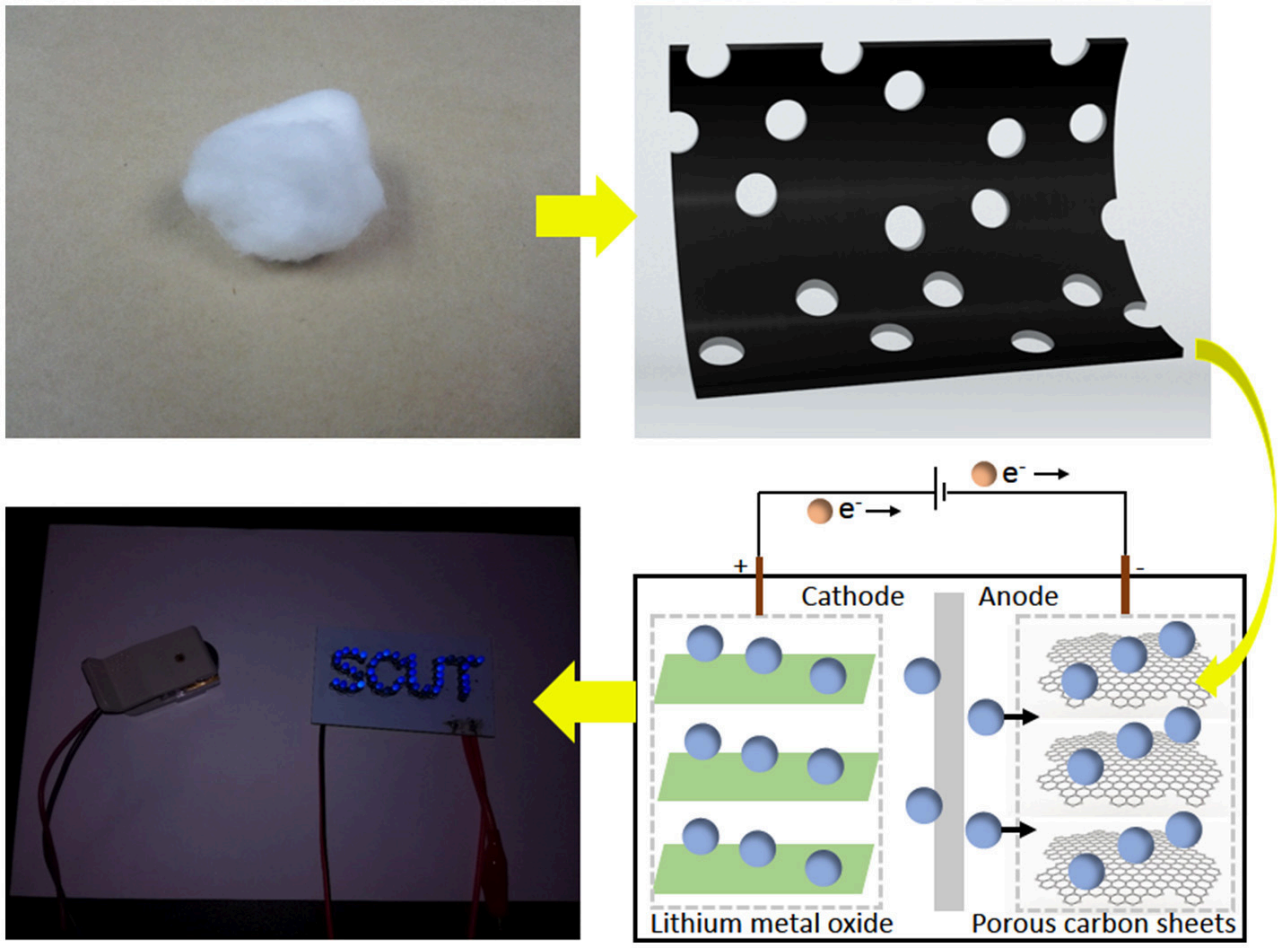
Schematic of the fabrication of porous carbon sheet anode form wasted cotton for Lithium-ion battery anode powering blue light emitting diode (LED).

Herein, we addressed the above mentioned issues by prepared high performance carbon-based anode materials using cotton as precursor, as shown in Figure 1. Highly porous carbon with large surface areas were prepared from cotton via s sample method. And the N/S-coped carbon were further obtained using thiourea as nitrogen and sulfur sources. When evaluated as anode materials for LIBs, these carbon materials exhibit outstanding rate capability and long-term cycling stability.

## Experimental

### Material preparation

1.5 g cotton was dipped in homogeneous Mg(NO_3_)_2_ solution (8 mol L^−1^, 20 ml), then dried in the oven. After that, the obtained cotton were annealed at 800°C under N_2_ atmosphere for 3 h with a heating rate of 5°C min^−1^. After cooling, the obtained cotton carbon (denoted as CC) were washed with 1 M HCl and distilled water several times, respectively, then dried in the oven.

To obtained the N, S co-doped carbon, the obtained CC were immersed in 100 ml thiourea solution with ratio of 10:1. After drying, the obtained powders were calcined at 800°C under N_2_ atmosphere for 3 h. The N, S co-doped cotton carbon (denoted as NS-CC) powders were obtained after cooling.

### Material characterizations

The XRD patterns of all samples were conducted on the Bruker D8 Advance (Germany) (Cu, Kα, λ = 1.5405 Å). Raman spectra were obtained on a JOBIN-Yvon HR800 Raman spectrometer. Morphology of the all samples were studied by SEM (FEI Quanta 200 FEG) and TEM (Tecnai G2 F20 S-TWIN, Japan). BET method and non-linear density functional theory (NLDFT) (ASAP 2020 Micromeritics) were applied to test the specific surface area and the pore size distribution.

### Electrochemical measurements

The samples were executed in CR2025 coin cells. The mingled ratio of sample CC, sample NS-CC and carbon black and poly (vinylidenedifluoride) were 7:2:1 (the loaded active electrode materials are about 0.5 mg cm^−2^). The mixture was coating on copper foil to prepare electrodes. Metal lithium boil as counter electrode and 1 M LiPF_6_ dissolved in ethylene carbonate (EC) and dimethyl carbonate (DMC) (1:1, v/v) as electrolyte. CV and EIS were conducted on a CHI660A electrochemical workstation. Galvanostatic charge/discharge and cycling performance were executed at 25°C based on the active electrode material corresponding specific capacity.

## Results and discussions

**Figure 2:**
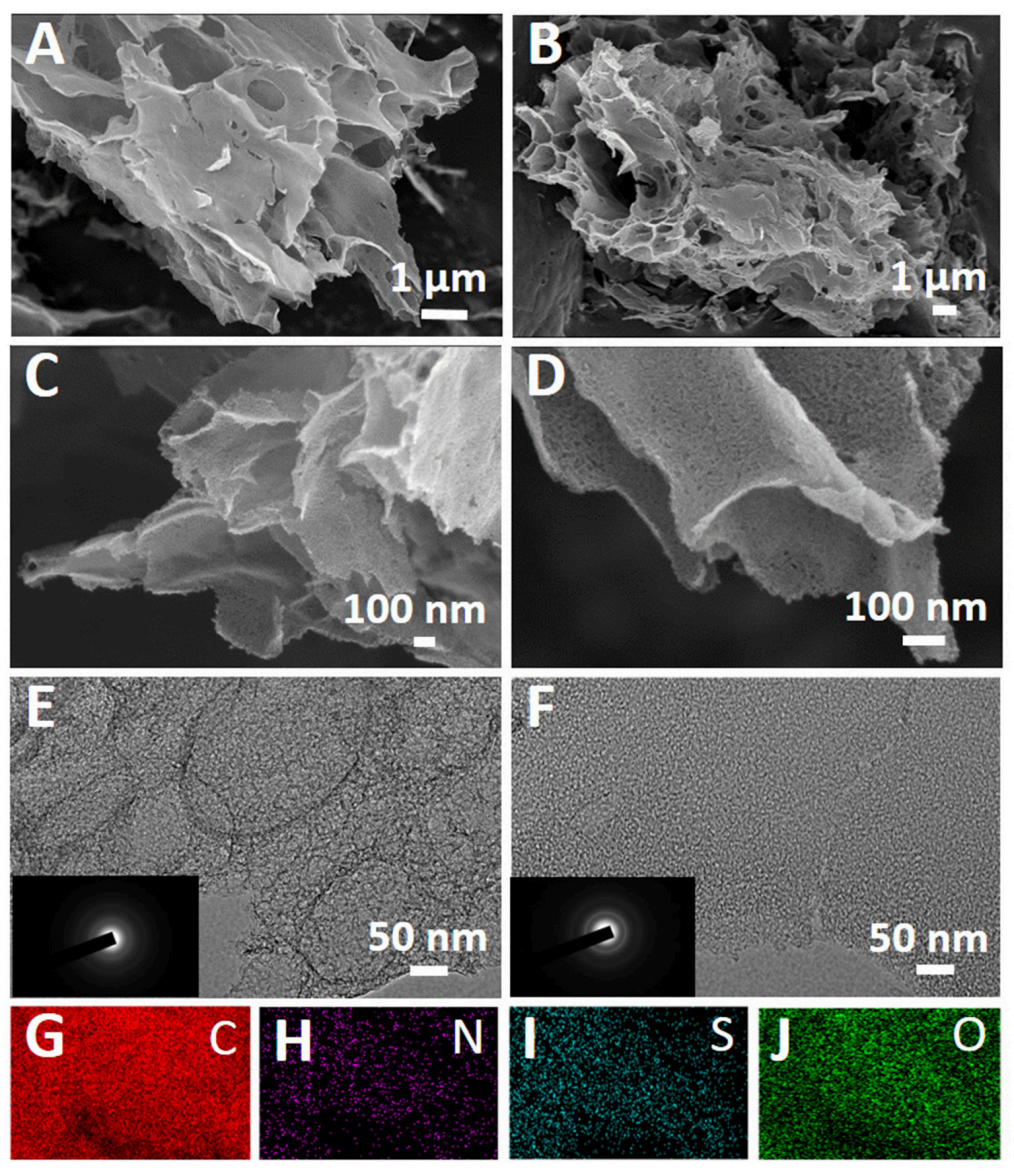
SEM images of **(A,C)** CC and NS-CC **(B,D)**; TEM images of **(E)** CC and **(F)** NS-CC (the inset of part is the SAED pattern); **(G–J)** EDS mapping of NS-CC.

The morphology of the all samples was characterized by SEM firstly. Figures 2A,C exhibit the SEM images of cotton carbon, which shows micron size bulk materials and composed of nanosheets. Figures 2B–D shows the SEM images of the cotton carbon after N and S co-doped, which exhibits similar morphology to cotton carbon. Moreover, cotton carbon and S, N co-doped cotton carbon with a large number of pores in the nanosheets according to the HRSEM (Figures 2C,D). On the other hand, EDS element mapping of S, N co-doped cotton carbon were investigated, which indicated that C, N, O, and S elements exist S, N co-doped cotton carbon. Furthermore, S and N elements evenly distributed (Figures 2G–J) in the carbon matrix. Therefore, the results indicated that the N and S elements were successfully doped into cotton carbon after heat treatment the carbon in presence of thiourea. The microstructure of CC and NS-CC was further studied by TEM, and the results are shown in Figures 2E,F. It clearly illustrates that the samples are amorphous carbon with nano/meso porous structure (Chen et al., 2014). Furthermore, typical selected area electron diffraction further proved that the carbon were amorphous, which corresponding to TEM results (Zhu and Akiyama, 2016).

**Figure 3:**
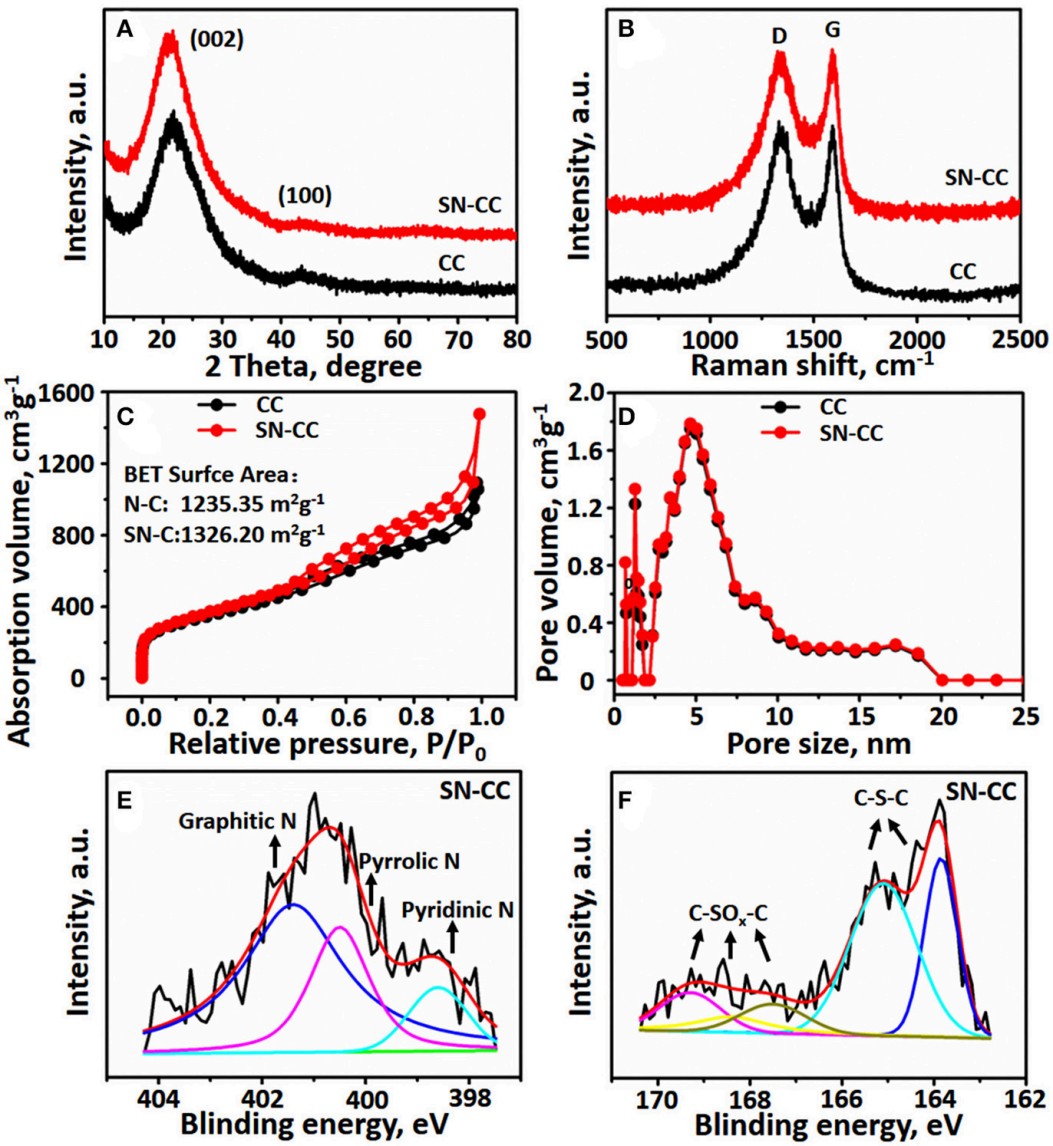
**(A)** XRD patterns **(B)** Raman spectrum **(C)** Nitrogen adsorption/desorption isotherm **(D)** Pore size distribution of CC and NS-CC. High-resolution scans of **(E)** N spectrum **(F)** S2p spectrum electrons of NS-CC.

The XRD patterns of the CC and NSCC are showed in Figure 3A. There are no obvious peaks for magnesium compounds in CC sample, which indicated that the Mg(NO_3_)_2_ or MgO are removed completely by washed with dilute hydrochloric acid. And two broad peaks at around 23 and 43° can be observed both at CC and NS-CC samples, which can be attributed to (002) and (100) graphitic planes, respectively (Hou et al., 2015; Chen et al., 2017). The Raman spectra of the CC and NS-CC are shown in Figure 3B, two peaks at around 1,361 and 1,596 cm^−1^ were obtained, which corresponding to the D band and G band for carbon materials, respectively (Li et al., 2015; Gao et al., 2017). Furthermore, the D band arises from edges, defects, and disordered carbon, whereas the G band is ascribed to sp^2^-hybridized carbon (Pan et al., 2017b). Therefore, a high I_D_/I_G_ band intensity ratio indicates the generation of large amounts of defects. The I_D_/I_G_ ratio for NS-CC is higher than that of CC, which indicated that more vacancies and defects generated by doping N and S atoms into the carbon material. More importantly, more vacancies and defects are beneficial for the transmission of Li-ion and offer more active site for Li storage, which resulting in improved electrochemical performance (Qie et al., 2015; Lu et al., 2017).

The XPS measurement was conducted to confirmed that the presence of N and S elements in NS-CC. As shown in Figures S1A,B, the survey spectrum for NS-CC and CC exhibit two predominant peak at around 284.8 and 532 eV can be observed, which can be assigned to C and O. It certified for the NS-CC that N and S atoms were successfully doped corresponding with EDS element mapping. In Figures S1C,D, the C 1s XPS spectrum for both CC and NS-CC can be deconvoluted into five peaks, which corresponding to C = C, C-C/C = N, C-O, C = O and π-π^*^ (Liu et al., 2017), respectively. The high resolution N 1s spectrum as shown in Figure 3E, the N 1s speak can be fitted by three component peaks at around 401.9, 400.5, and 398.3 eV, which can be ascribed to graphitic N, pyrrolic N and pyridinic N, respectively (Ou et al., 2015). As for high resolution of S 2p spectrum (Figure 3F), there were five peaks attributed to -C-S-C- bond and -C-SO_X_-C- bond. Therefore, these results indicated that the N and S has been successfully incorporated into the carbon structure of NS-CC. And the content of N and S in NS-CC were confirmed for 3.0 and 1.4%, respectively. The heteroatoms N and S can effectively enlarge the interlayer space because of their lager radius than C atom, resulting in forming the defects and providing more active sites for Li-ions on the carbon materials (Xu et al., 2015, 2016; Xiong et al., 2016). Moreover, pyridinic N and quaternary N are favorable for Li^+^ and electrons and the doped S in the carbon materials can participate in the redox reactions contribute to the reversible capacity (Ma et al., 2018).

The nitrogen adsorption/desorption isotherms and the pore size distribution of CC and NS-CC are shown in Figures 3C,D. As shown in Figure 3C both of the two samples showed type IV isotherms (Islam et al., 2017). The BET specific surface area of CC and NS-CC are 1235.35 and 1326.20 m^2^ g^−1^, respectively. The BET specific surface areas of NS-CC increased compare than the CC after N and S atoms doping. In addition, the highly porous structure of the two samples were further evaluated by Barrett-Joyner-Halenda (BJH) calculations (Figure 3D). It can be seen that the two samples exhibit a broad pore size distribution, and the pore size of the two samples were centered at around 2 and 5 nm, respectively. Therefore, NS-CC exhibits larger specific surface area which can provide more active sites for lithium ion storage. Moreover, the highly porous structure of the two samples can greatly shorten the diffusion distance of both electrons and ions, which resulting in improved rate performance (Hao et al., 2014).

**Figure 4:**
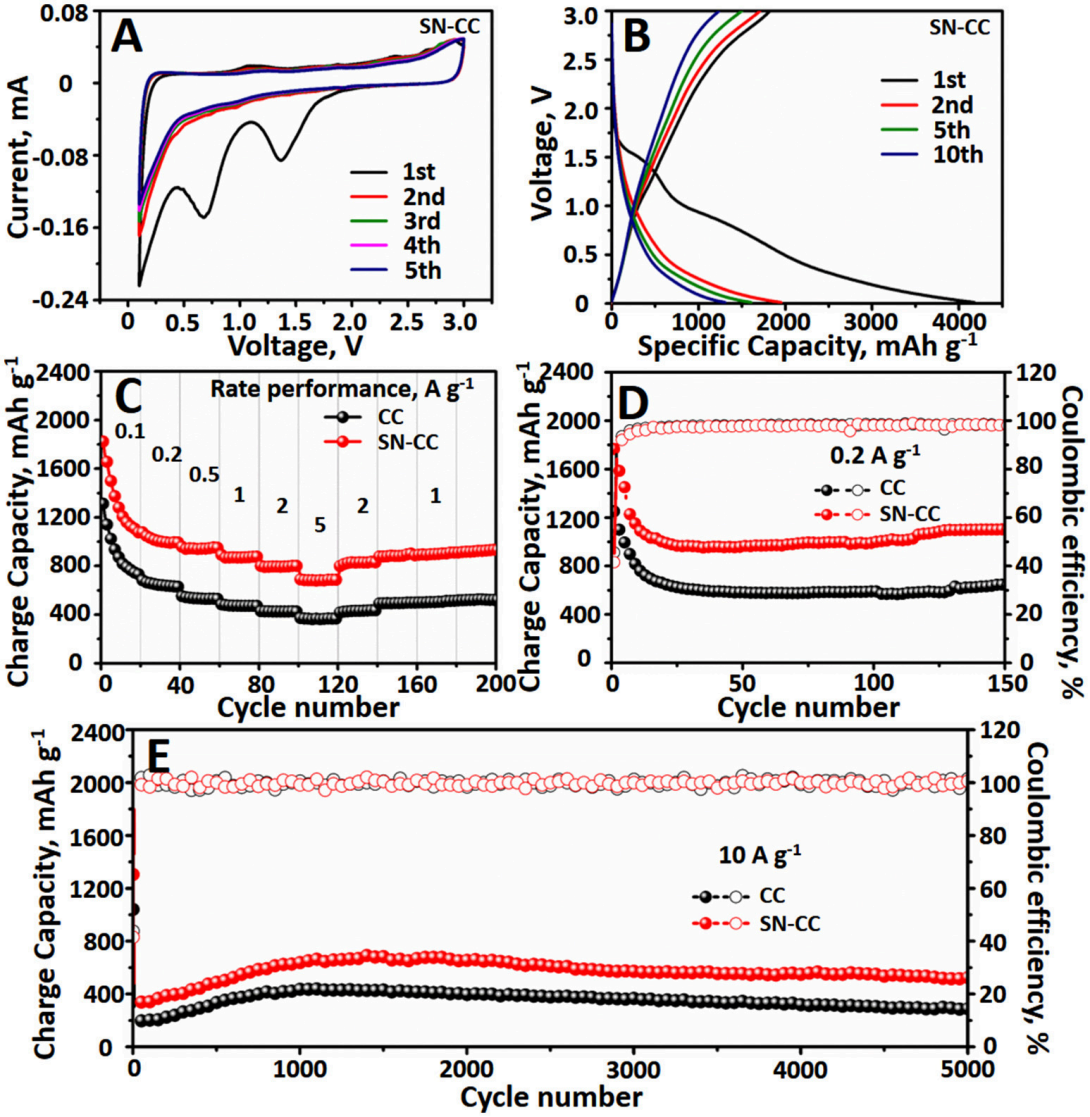
**(A)** CV curves and **(B)** first 10 cycles of charge-discharge profiles of NS-CC; **(C)** Rate performance and **(D)** cyclic performance at 0.2 A g^−1^; **(E)** long-term cyclic performance at 10 A g^−1^ of CC and NS-CC.

The electrochemical performance of CC and NS-CC was first measured by cyclic voltammetry (CV) with voltage range of 3.0–0.01 V. Figure 4A exhibits the CV curves of the NS-CC sample. During the first discharge cycle, three cathodic peaks at around 1.4 and 0.65 V can be obtained and disappeared in the subsequent cycles, which corresponding to formation of a solid-electrolyte interphase (SEI) film (Wang et al., 2011; Jiang et al., 2013) as well as some irreversible and side reactions associated with the decomposition of electrolyte (Yoshio et al., 2000). Moreover, the CV profiles almost overlapped after the initial scanning cycle, which indicates that the structural stability of the NS-CC electrode during the subsequently cycling. On the other hand, the CV curves of CC electrode (Figure S2A) are similar to the NS-CC electrode.

The charge and discharge profiles of CC and NS-CC electrode at 0.1 A g^−1^ with cutoff voltage window of 0.01–3.0 V were studied. As shown in Figure 4B and Figure S2B, two plateaus at around 1.4 and 0.65 V can be observed during the first discharge process, which corresponding to the forming of SEI layer and decomposition of the electrolyte, in good agreement with the above CV results. The first discharge capacity of CC and NS-CC are as high as 3,275.1 and 4,179.1 mA h g^−1^, while the initial reversible capacity are only 1,512.3 and 1,823.3 mA h g^−1^, corresponding to low initial Coulombic efficiency of 46.2 and 43.6%, respectively. The large irreversible capacity loss for the CC and NS-CC electrode can be ascribed to formation of a SEI layer on the relatively large specific surface area (Jiang et al., 2013). Furthermore, a mass of reduction of oxygen functionalities on the carbon materials surface (Bhattacharjya et al., 2014) and reduction of electrolyte components on the active electrode of the CC and NS-CC electrode (Hu et al., 2007) also contributed to the irreversible capacity loss.

The cycling performance of the CC and NS-CC electrode were evaluated at 0.2 A g^−1^ (Figure 4D). NS-CC electrode exhibits excellent cycling stability and a high reversible capacity of 1101.1 mA h g^−1^ can be observed after 150 cycles, but for CC electrode, a lower reversible capacity of 637.1 mA h g^−1^ can be obtained. Rate performance is very important for LIBs, especially application in electric vehicles. Therefore, the rate performance of the samples were evaluated at various current densities from 0.1 to 5.0 A g^−1^. As seen in Figure 4C, NS-CC electrode can deliver reversible capacities of 1,443, 1,035, 954, 884, 802, and 689 mA h g^−1^ at 0.1, 0.2, 0.5, 1.0, 2.0, and 5.0 A g^−1^, respectively. In contrast, the reversible capacities are 1,020, 655, 541, 478, 427, and 370 mA h g^−1^ at 0.1, 0.2, 0.5, 1.0, 2.0, and 5.0 A g^−1^ for CC electrode. Obviously, the NS-CC electrode exhibits excellent rate capability. Therefore, the long-term cycling at high current density was also tested for NS-CC electrode, the results as shown in Figure 4E. NS-CC electrode can deliver a high reversible capacity of 531.2 mA h g^−1^ can be obtained even after 5,000 cycles at 10.0 A g^−1^. However, CC electrode only delver a lower reversible of 283 mA h g^−1^ after 5,000 cycles at the same current density. Therefore, NS-CC electrode exhibits excellent rate performance and long-term cycling stability, which shows better electrochemical performance than previous reported carbon-based materials, as shown in Table S1. As a result, The NS-CC electrode delivered amazing electrochemical performance especially with ultrahigh specific capacity and rate capability which can be given rise to the following reasons: (1) The carbon materials interlayer spacing are expanded by N and S successfully co-doping which benefit Li-ions diffusion. (2) The marked large surface of carbon materials offer plentiful micropores and mesopores structure shorten the diffusion distance, sufficient contact between electrolyte and electrode and active sites for lithium ion storage. (3) The ample pyridinic N, pyrrolic N and -S-C-S- covalent bonds built adequate active sites to improve surface capacity contribution (Xia et al., 2017).

The electrochemical impedance spectroscopy for CC and NS-CC electrode were further investigated to understand the significantly improved electrochemical performance. As shown in Figure S3, the Nyquist plots of CC and NS-CC electrode have shown the typical characteristics of one semicircle and a sloping straight line (Liu et al., 2016). The diameter of the semicircle is reduced in the plots of the NS-CC electrode compared with that of the CC electrode, indicating the decreased charge-transfer resistance at the electrode/electrolyte interface after doping of N, S atoms into the carbon. On the other hand, the charge transfer resistance presents a decreasing trend along with the cycles for both CC and NS-CC electrode, which due to formation of stable SEI film and the process of activation after cycling (Pan et al., 2016).

**Figure 5:**
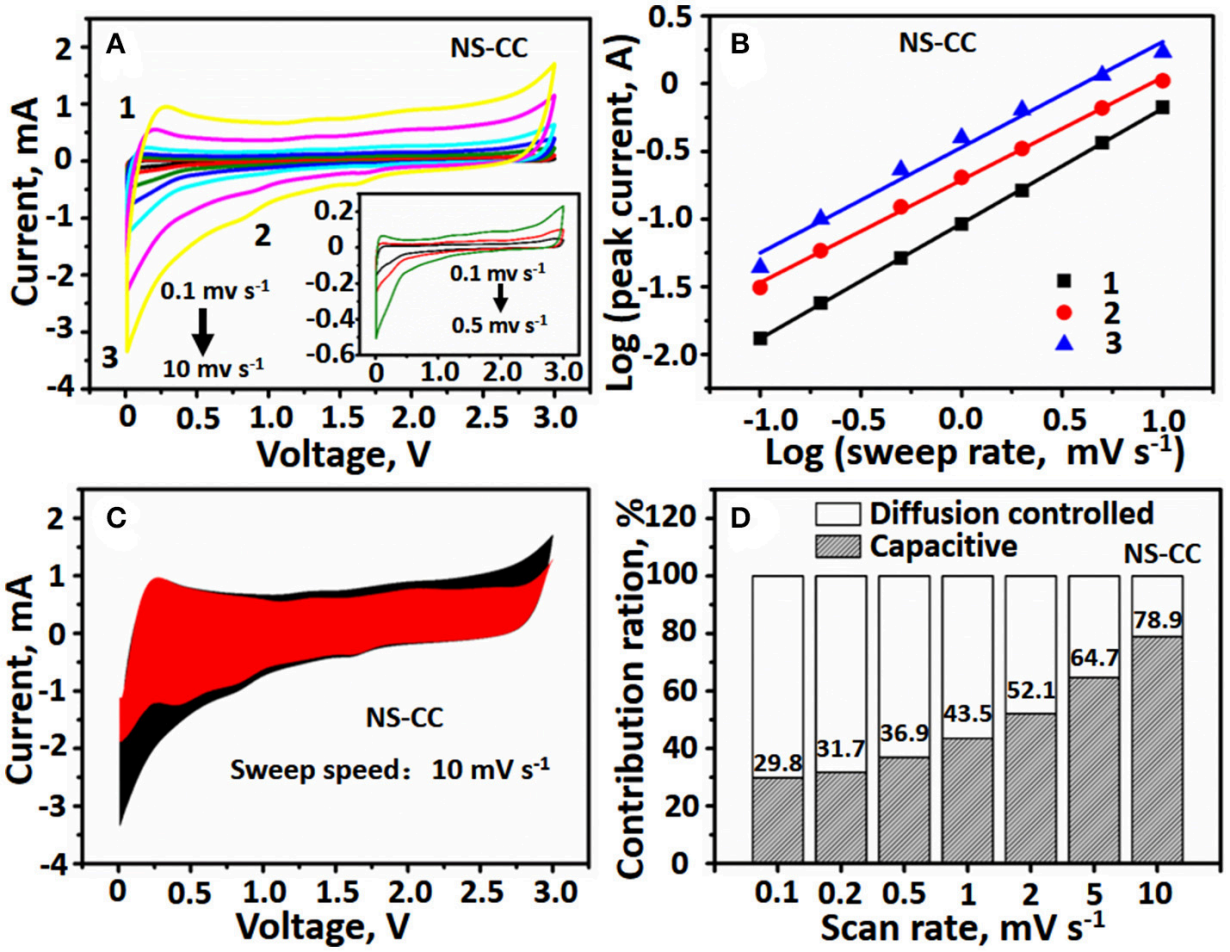
**(A)** CV curves measured between 0.01 and 3.0 V at various scan rate from 0.1 to 10 mV s^−1^. **(B)** The *b*-value determined by using the relationship between peak current and scan rate. **(C)** CV curve with the pseudocapacitive fraction shown by red and diffusion shown by black at a scan rate of 10 mV s^−1^. **(D)** Bar chart showing the percentage of pseudocapacitive contribution at vs. scan of NS-CC.

In order to further understand the high-rate performance, the capacitive behavior of the NS-CC and CC electrode were investigated and their kinetics were also analyzed with CV measurements. Figure 5A and Figure S4A show the CV curves of NS-CC electrode at various scan rates ranging from 0.1 to 10 mV s^−1^. All of the curves display a similar shape, two cathodic peaks and one anodic peak are evidently on each curve. The peak current is not proportional to the square root of the sweep rate (*v*), indicating that the charge/discharge process is comprised of faradic and non-faradic processes. According to the equation of the relationship of *i* and *v*:

(1) $$\begin{array}{r} i=av^{b} \end{array}$$

or

(1)′ $$\begin{array}{l} \;\log\left(i\right)=b\times \log\left(v\right)+\log{\left(a\right)}^{\prime } \end{array}$$

Here, a and b are constants. The process is an ionic diffusion controlled behavior when *b*-value is equal to 0.5, while is Li^+^ capacitive behavior when *b*-value is equal to 1.0. Figure 5B presents log(*i*)-log(*v*) plots for NS-CC electrode on the CV curves at peak 1, 2 and 3 potentials and the *b*-value are 0.84, 0.75 and 0.77, respectively. And for CC the *b*-value are 0.75, 0.71, and 0.97, respectively (Figure S4B). Therefore, It can be seen that all these values of b indicate fast kinetics resulting from the pseudocapacitive effect. Moreover, To quantify the pseudocapacitive contribution, we can divide the current response i at a fixed potential V into pseudocapacitive (k_1_*v*) and diffusion-controlled contributions (${\text{k}}_{2}v^{0.5}$) by following equation (Muller et al., 2015).

$$\begin{array}{l} i\left(V\right)=k_{1}v+K_{2}v^{1/2} \end{array}$$

By calculating both k1 and k2 constants, the overall contribution of pseudocapacitor at various scan rates can be obtained. The detail pseudocapacitive contribution of NS-CC and CC electrode at 10 mV s^−1^ as shown in Figure 5C and Figure S4C, in which 78.9% as capacitive (red region). Therefore, all contribution ratios of the capacitive capacity at scan rates of 0.1, 0.2, 0.5, 1, 2, and 5 mV s^−1^ were also obtained. Figure 5D and Figure S4D shows contributions of the pseudocapacitive behaviors at various scan rates. The proportion of capacitive contribution for NS-CC electrode are 29.8, 31.7, 36.9, 43.5, 52.1, 64.7, and 78.9% at 0.1, 0.2, 0.5, 1, 2, 5, and 10.0 mV s^−1^, respectively. Furthermore, the contribution for CC electrode are 27.1, 28.2, 32.3, 41.3, 50.1, 62.6, and 75.6% at 0.1, 0.2, 0.5, 1.0, 2.0, 5.0, and10.0 mv s^−1^. As a result, the capacitive contribution for CC is smaller than the NS-CC electrode, which can attribute to the NS-CC electrode with larger specific area after N and S atoms co-doping, resulting in enhanced pseudocapacitive contribution (Augustyn et al., 2014; Chao et al., 2016).

## Conclusion

In summary, highly porous carbon were prepared by using cotton as precursor with a sample method. Subsequently, the sulfur-nitrogen co-doped carbon were obtained via heat treatment the carbon in presence of thiourea, which can induce the defects and the expanded interlayer of the carbon. Therefore, the expanded interlayer and defects can reduce the diffusion distance of Li ions as well as offer more active sites for lithium storage. As a result, S, N co-doped carbon exhibits excellent electrochemical performance when evaluated as anode materials for Lithium-ion materials. The as-prepared S, N co-doped carbon can deliver a high reversible capacity of 546.4 mA h g^−1^ even after 5,000 cycles at 10 A g^−1^. Moreover, excellently rate capability also can be observed, a high capacity of 600 mA h g^−1^ can be obtained at 5.0 A g^−1^. This superior lithium storage performance of S, N co-doped carbon make it as a promising low-cost and sustainable anode material for lithium ion batteries.

## Author contributions

JX conducted the experiments CY is the supervisor of this research work. JX and QP helped writing. JX, FZ, XX, DH, and CH performed the characterization and data analysis. All authors involved the analysis of experimental data and manuscript preparation.

### Conflict of interest statement

DH and CH were employed by company Jiangsu Key Lab of Silicon Based Electronic Materials. The other authors declare that the research was conducted in the absence of any commercial or financial relationships that could be construed as a potential conflict of interest.
