# Supplementary material for: N/S Co-doped Carbon Derived From Cotton as High Performance Anode Materials for Lithium Ion Batteries
Source: Front Chem. 2018 Apr 26;6:78. doi: 10.3389/fchem.2018.00078 (PMC5932144; doi:10.3389/fchem.2018.00078)
Supplement: Supplementary file 1 [file Presentation1.PDF]

Supplementary Material

Sustainable N/S co-doped Carbon Derived from Cotton as high performance anode materials for lithium ion batteries

Jiawen Xiong [1], Qichang Pan [1], Fenghua Zheng [1], Xunhui Xiong [1], Chenghao Yang [1*], Dongli Hu [2], Chunlai Huang [2]

*** Correspondence:** Corresponding Author: email@uni.edu

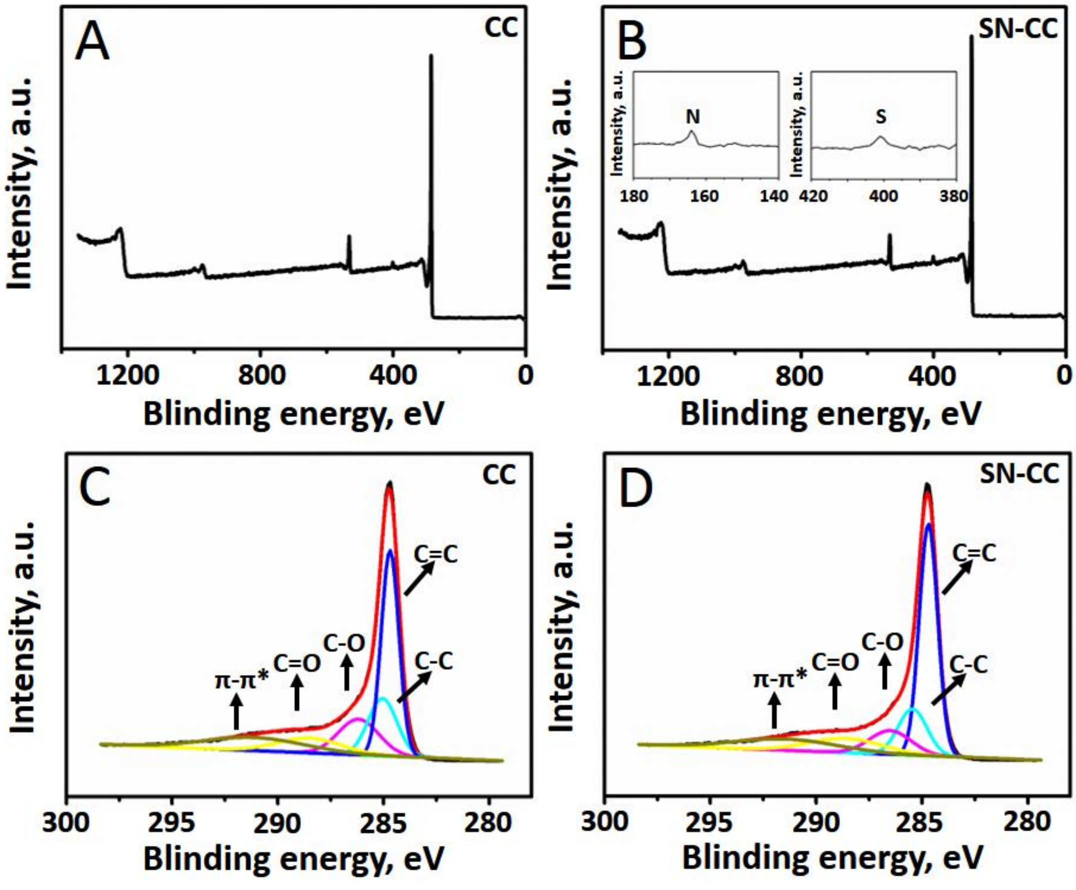

Figure S1. XPS spectrum survey of CC (A) and NS-CC (B). XPS C1s of CC (C) and NS-CC (D).

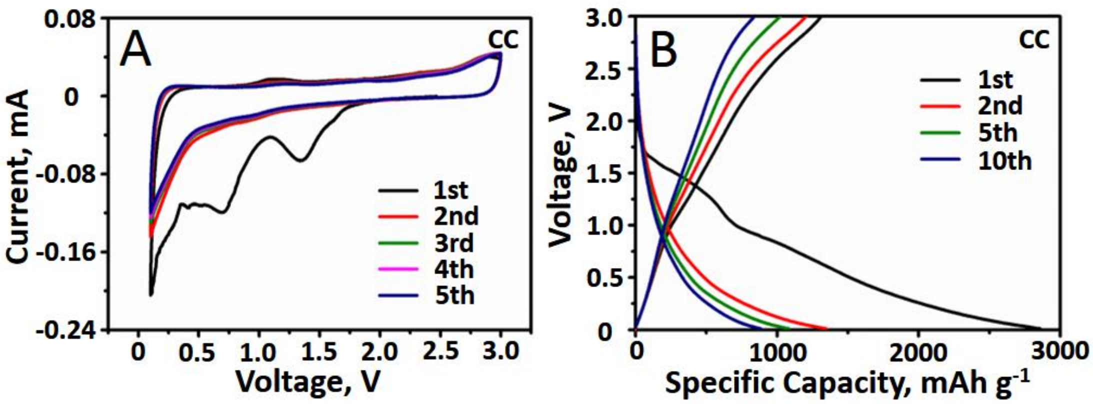

Figure S2. (A) CV curves and (B) first ten cycles of charge-discharge profiles of CC.

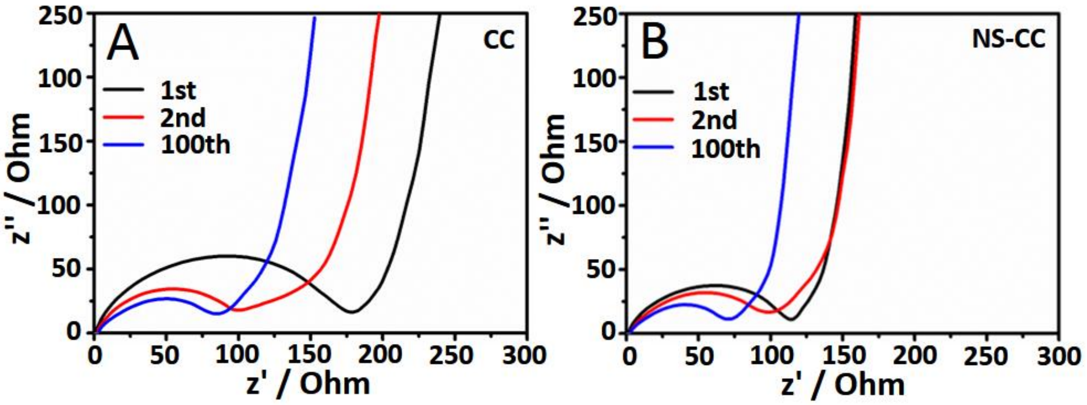

Figure S3. Electrochemical impedance spectra of CC (A) and NS-CC (B).

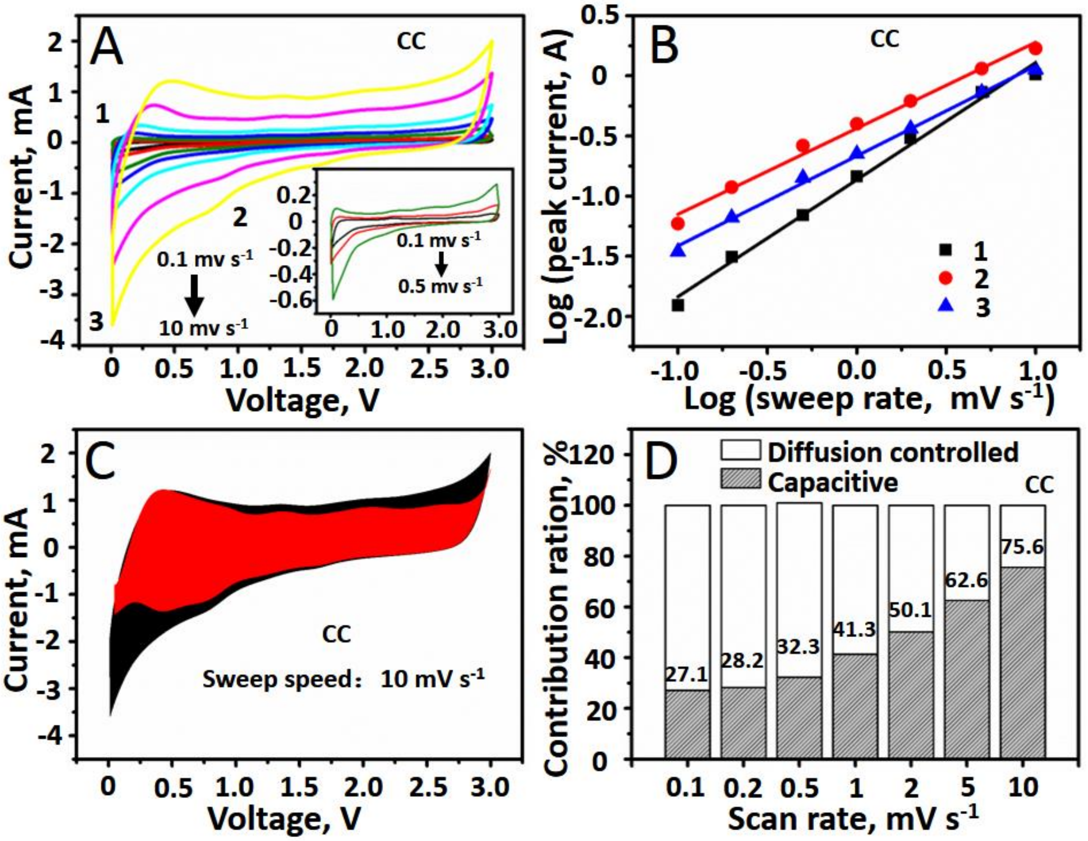

Figure S4. (A) CV curves measured between 0.01-3.0V at various scan rate from 0.1 to 10 mV s^{-1}. (B)The b-value determined by using the relationship between peak current and scan rate. (C) CV curve with the pseudocapacitive fraction shown by red and diffusion shown by black at a scan rate of 10 mV s^{-1}. (D) Bar chart showing the percentage of pseudo capacitive contribution at versus scan of CC.

Table S1. Cycling stability and rate performance comparison of Lithium-ion batteries (LIBs) carbon-based anode materials.

Materials	Sources	Cycling Stability (mAh g^{-1})	Rate Performance (mAh g^{-1})	References
graphite	Banana peels	1460 at 0.05 A g^{-1} after 300 cycles	180 at 10.0 A g^{-1}	Lotfabad et al., 2014
3D hierarchical porous carbon	Lignin	386 at 0.5 A g^{-1} after 560 cycles	268 at 1.0 A g^{-1}	Zhang et al., 2015
carbon nanofibers / nanosheets	Cornstalks	481 at 2.0 A g-1 after 400 cycles<br>592 at 0.1 A g^{-1} after 200 cycles	454 at 3.0 A g^{-1}	Wang et al., 2015
carbon nanoparticles	Coconut oil	577 at 2.0 A g^{-1} after 20 cycles	295 at 1.0 A g^{-1}	Gaddam et al. 2016
Boron and Nitrogen cual-doped 3D carbon nanofibers	Carbon fibers	430 at 2.0 A g^{-1} after 5000 cycles	375 at 5.0 A g^{-1}1<br>272 at 10.0 A g^{-1}	Xia et al., 2017
Sulfur doped graphene	graphene	870 at 0.1 A g^{-1} after 160 cycles	433 at 2.0 A g^{-1}	Yun et al., 2014
heteroatom doped porous carbon	Human hair	1325 at 0.1 A g^{-1} after 200 cycles<br>752 at 1.0 A g^{-1} after 200 cycles	470 at 5.0 A g^{-1}<br>205 at 10.0 A g^{-1}	Ou et al., 2014
N-doped porous Carbon	paraffin oil	1275 at 0.186 A g^{-1} after 250 cycles<br>310 after at 3.7 A	413 at 1.86 A g^{-1}	Lu et al., 2017

		g^{-1} after 1500 cycles		
N-doped porous Carbon	Covalent-organic<br>-frameworks	488 at 0.1 A g^{-1}1 after 100 cycles	143 at 5.0 A g^{-1}	Zhang et al., 2017
N-doped porous carbon	Garlic peel	540 at 0.1 A g^{-1} after 100 cycles	145 at 8.0A g^{-1}	Selvamani et al., 2016
Sulfur and Nitrogen dual-doping carbon materials	Broad beans shells	261.5 at 0.37 A g^{-1} after 100 cycles	-	Xu et al., 2016
N-rich Carbon	Wheat straw	976 at 0.37 A g^{-1} after 300 cycles<br>659 after at 3.7A g^{-1} after 300 cycles	664 at 3.7A g^{-1}	Chen et al., 2014
**CC**	**Cotton**	**637.1 at 0.2 A g^{-1} after 150 cycles**<br>**283 cycles at 10.0 A g^{-1} after 5000**	**427 at 5.0 A g^{-1}**	**This work**
**NS-CC**	**Cotton**	**1101.1 at 0.2 A g^{-1} after 150 cycles**<br>**531.2 cycles at 10.0 A g^{-1} after 5000**	**689 at 5.0 A g^{-1}**	**This work**

Zhang, W., Yin, J., Lin, Z., Lin, H., Lu, H., Wang, Y., Huang, W. (2015). Facile preparation of 3D hierarchical porous carbon from lignin for the anode material in lithium ion battery with high rate performance. Electrochimica Acta 176, 1136-1142. DOI: 10.1016/j.electacta.2015.08.001

Wang, S., Xiao, C., Xing, Y., Xu, H., Zhang, S. (2015). Carbon nanofibers/nanosheets hybrid derived from cornstalks as a sustainable anode for Li-ion batteries. J. Mater. Chem. A 3, 6742-6746. DOI: 10.1039/c5ta00050e

Gaddam, R. R., Yang, D., Narayan, R., Raju, K., Kumar, N. A., Zhao, X. S. (2016). Biomass derived carbon nanoparticle as anodes for high performance sodium and lithium ion batteries. Nano Energy 26, 346-352. DOI: 10.1016/j.nanoen.2016.05.047

Yun, Y.S., Le, V.-D., Kim, H., Chang, S.-J., Baek, S.J., Park, S., Kim, B.H., Kim, Y.-H., Kang, K., and Jin, H.-J. (2014). Effects of sulfur doping on graphene-based nanosheets for use as anode materials in lithium-ion batteries. J. Power Sources 262, 79-85. DOI: 10.1016/j.jpowsour.2014.03.084

Ou, J., Zhang, Y., Chen, L., Yuan, H., and Xiao, D. (2014). Heteroatom doped porous carbon derived from hair as an anode with high performance for lithium ion batteries. RSC Adv. 4, 63784-63791. DOI: 10.1039/c4ra12121j

Zhang, X., Zhu, G., Wang, M., Li, J., Lu, T., and Pan, L. (2017). Covalent-organic-frameworks derived N-doped porous carbon materials as anode for superior long-life cycling lithium and sodium ion batteries. Carbon 116, 686-694. DOI: 10.1016/j.carbon.2017.02.057

Selvamani, V., Ravikumar, R., Suryanarayanan, V., Velayutham, D., and Gopukumar, S. (2016). Garlic peel derived high capacity hierarchical N-doped porous carbon anode for sodium/lithium ion cell. Electrochimica Acta 190, 337-345. DOI: 10.1016/j.electacta.2016.01.006
